# Paired related homeobox 1 attenuates autophagy via acetyl‐CoA carboxylase 1‐regulated fatty acid metabolism in salivary adenoid cystic carcinoma

**DOI:** 10.1002/2211-5463.13367

**Published:** 2022-03-29

**Authors:** Jie Gao, Kangjie Ma, Li Zhang, Tao Li, Baodong Zhao, Yaping Jiang

**Affiliations:** ^1^ 235960 Department of Oral Implantology The Affiliated Hospital of Qingdao University China; ^2^ 235960 School of Stomatology of Qingdao University China; ^3^ 235960 Department of Joint Surgery The Affiliated Hospital of Qingdao University China

**Keywords:** acetyl‐CoA carboxylase 1, autophagy, fatty acid metabolism, invasion, metastasis, paired related homeobox 1

## Abstract

Autophagy can affect the invasion and metastasis of carcinoma. Our previous study has shown that invasion and epithelial‐mesenchymal transition in salivary adenoid cystic carcinoma (SACC) can be promoted by the metabolic reprogramming of free fatty acids (FFAs). However, the effect of FFA metabolism on autophagy in SACC remains unknown. In this study, we showed that overexpression of paired related homeobox 1 (PRRX1) reduced the number of autophagosomes and decreased the expression of *LC3* and *Beclin‐1* in SACC patients and SACC‐83 cells *in vitro*. Moreover, PRRX1‐mediating FFA reprogramming triggered to autophagy via regulating acetyl‐CoA carboxylase 1 (ACC1), leading to invasion and migration in SACC.

## Graphical abstract

Overexpression of paired related homeobox 1 (PRRX1) reduces the number of autophagosomes and decreases the expression of *LC3* and *Beclin‐1* in salivary adenoid cystic carcinoma patients (SACC) and SACC cells *in vitro*. Moreover, free fatty acids reprogramming mediated by PRRX1 triggers autophagy via regulation of acetyl‐CoA carboxylase 1, thereby accelerating invasion and migration in SACC.

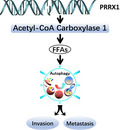

**Abbreviations**
ACC1: acetyl‐CoA carboxylase 1
AMPK: adenosine monophosphate activated protein kinase
BCA: bicinchoninic acid
EMT: epithelial‐mesenchymal transition
FFAs: free fatty acids
GAPDH: glyceraldehyde phosphate dehydrogenase
GC/MS: gas chromatography mass spectrometry analysis
LC3B: light‐chain 3 beta
NT: normal tissues
OA: oleic acid
PRRX1: paired related homeobox 1
RPMI‐1640: Roswell Park Memorial Institute‐1640
RT‐qPCR: reverse transcription quantitative polymerase chain reaction
SACC: salivary adenoid cystic carcinoma
TEM: transmission electron microscopy

As one of the most common salivary gland tumors, salivary adenoid cystic carcinoma (SACC) has a poor long‐term prognosis [1, 2, 3, 4]. Metastasis and perineural invasion are the major risk factors for the long‐term poor prognosis of SACC [5, 6]. Due to the poor understanding of the mechanism underlying the SACC progression, no effective therapy has been provided [7]. Therefore, it would be extraordinarily significant to understand the molecular mechanism involved in the invasion and metastasis of SACC to develop more effective diagnostic and therapeutic strategies [8, 9].

The reprogramming of lipid metabolism can not only generate energy source by β‐oxidation but also provide phospholipids for the membrane synthesis of cancer cells, which can aid the proliferation and invasion of tumor cells. Besides liver cells and adipocytes, the *de novo* synthesis level of fatty acid is relatively low in normal cells. However, it has been reported that the pathway of *de novo* synthesis of fatty acid is selectively activated in the malignant cells of breast, lung, prostate, ovarian tumors, etc. [10, 11, 12, 13, 14]. As a rate‐limiting enzyme for the *de novo* synthesis of lipids, acetyl‐CoA carboxylase 1 (ACC1) plays a crucial role in lipid metabolism. By reducing the ACC1 activity, the levels of total acetyl‐CoA, as well as migration, and invasion in breast cancer cells are elevated [15]. It has been reported that the chronic stress and morphologic changes in the endoplasmic reticulum was caused by the alterations of *de novo* fatty acid synthesis compromising the regulation of autophagy, including the formation of autophagy associated gene aberration and drastically impaired autophagosome biogenesis in yeast cells [16]. However, Gross has reported that ACC1‐dependent lipogenesis can promote autophagy in yeast strains [17]. Nevertheless, it remains largely unexplored whether ACC1 affects autophagic activity in humans.

Paired related homeobox 1 (PRRX1) is an important transcription factor of embryonic development and epithelial‐mesenchymal transition (EMT) of the tumor, which is highly expressed in breast, lung, colon tumors, etc. PRRX1b is associated with the invasion of pancreatic cancer cells, while PRRX1a is highly correlated to migration. The two subtypes are highly expressed in metastatic pancreatic cancer cells [18]. It has been found that there is overexpressed *PRRX1* in abdominal subcutaneous adipose tissue of obese patients by DNA microarray analysis [19]. PRRX1 can play a role on lipid metabolism and induce the release of free fatty acids (FFAs), promoting the development of type II diabetes [20]. According to our previous study, FFAs are related to invasion and EMT in SACC [21]. Until now, the mechanism of PRRX1 in the lipid metabolic reprogramming and metastasis of cancers is still unclear.

Autophagy affects the invasion and metastasis of carcinoma [22]. Autophagy can suppress the metastasis by preventing EMT [23] and promote the metastasis by contributing to tumor‐stromal metabolic coupling [24]. Recent studies have used Beclin‐1 and LC3B as significant biochemical markers for autophagy [25, 26]. The findings—alterations in the expression of LC3B and Beclin‐1 in cancer cells—suggest that autophagy may directly influence the biological behaviors of the carcinomas [27]. Additionally, autophagy can impair metastasis in human SACC [28]. Up to now, it remains unclear whether PRRX1 affects autophagic activity in SACC.

## Materials and methods

### Cell culture

SACC‐83 cells were donated by the Laboratory of Stomatology, the Affiliated Hospital of Qingdao University, and the Laboratory originally purchased SACC‐83 cells from Shanghai Life Science College Cell Resource Center, Chinese Academy of Sciences, China. The cell STR reports are shown in Fig. S1 and Table S1. Cells were cultured in RPMI‐1640 medium supplemented with 10% FBS and antibiotic cocktail consisting of penicillin and streptomycin (Solarbio) at 37 °C in a humidified atmosphere containing 5% CO_2_.

### Immunohistochemistry

Freshly collected tissue samples were obtained from the 32 SACC patients and the 32 patients of benign salivary gland tumor underwent surgery, who were registered between November 2016 and October 2020 in the Department of Oral and Maxillofacial Surgery, the Affiliated Hospital of Qingdao University. The experimental protocols were approved by the Institutional Ethics Committee of the Affiliated Hospital of Qingdao University. Every patient was understanding and signed separate informed consent forms for sampling and molecular analysis. The study methodologies conformed to the standards set by the Declaration of Helsinki. None of the SACC patients had undergone preoperative radiotherapy or chemotherapy. The tissue samples were fixed in 10% formaldehyde and embedded in paraffin. Sections were prepared, and immunohistochemical staining was conducted using a conventional protocol. Briefly, sections were incubated with anti‐PRRX1 (1 : 80; Novus Biologicals, Centennial, CO, USA), anti‐ACC1 (1 : 500; Proteintech, Rosemont, IL, USA), and anti‐Beclin‐1 (1 : 500; Proteintech) for 2 h, followed by incubation with secondary antibody. Then, colorimetric detection was performed using a DAB kit (ZSGB BIO).

### Lentivirus transfection and confirmation of transfection

A lentiviral vector carrying *PRRX1* gene was successfully constructed using 293T cells. Cells were transfected with *PRRX1* overexpression lentiviral vector harboring a luciferase reporter gene (Genechem Corporation, Shanghai, China) for 48 h, followed by the transduction of lentivirus in the supernatant into SACC‐83 cells. The lentivirus transfection efficiency was determined based on the fluorescence intensity of luciferin using a fluorescence microscope.

### Real‐time RT‐PCR

Trizol reagent (Invitrogen) was adopted to extract total RNA. Purified RNA was reversely transcribed into cDNA. Briefly, after an initial denaturation step at 95 °C for 15 min, the amplifications were carried out with 45 cycles at a melting temperature of 94 °C for 15 s, an annealing temperature of 60 °C for 25 s, and an extension temperature of 72 °C for 15 s. GAPDH was selected as a housekeeping gene. The relative expressions of target genes were calculated using the 2^−ΔΔCt^ method. The primer sequences of the target genes were as follows:

*BECLIN 1*, forward primer: 5′‐TGCGACAGTCTCTCCGTGC‐3′, reverse primer: 5′‐GGCCACTTCCAGAGCCTTTC‐3′

*LC3B*, forward primer: 5′‐AGAGCGATACAAGGGTGAGAAG‐3′ and reverse primer: 5′‐AGAAGGCTTGGTTAGCATTGAG‐3′

*GAPDH* forward primer: 5′‐ATGGGGAAGGTGAAGGTCG‐3′ and reverse primer: 5′‐TAAAAGCAGCCCTGGTGSACC‐3′.

### Western blotting analysis

The samples prepared for western blot analysis based on the instruction of whole protein extraction kit (KeyGEN). The whole protein extracted was quantified by the bicinchoninic acid protein assay kit (Beyotime). Briefly, equal amounts of proteins (25 μg) were subjected to sodium dodecyl sulfate‐polyacrylamide gel electrophoresis on 10% gels and then electro‐transferred onto polyvinylidene difluoride membranes (Millipore, Darmstadt, Germany). Nonspecific bindings were blocked using 4% bovine serum albumin (BSA). Subsequently, membranes were incubated with rabbit anti‐PRRX1 (1 : 1000; Abcam, Waltham, MA, USA), rabbit anti‐LC3B (1 : 1000; Proteintech), rabbit anti‐Beclin‐1 (1 : 1000; Proteintech), rabbit anti‐ACC1 (1 : 1000; Proteintech), or rabbit anti‐GAPDH (1 : 2000; Elabscience, Wuhan, China) at 4 °C overnight. Immunoreactive bands were visualized via using the enhanced chemiluminescence kit (Millipore) according to the instructions.

### Transmission electron microscopy (TEM)

SACC‐83 cells were centrifuged at 500 × ***g*** for 6 min at 4 °C, and then, the supernatant was discarded. Cell pellets were resuspended in 2.5% glutaraldehyde at 4 °C for 10 h. The fixed cells were then exposed to 1% OsO_4_ at room temperature for 1 h. The cells were dehydrated in a graded series of acetone and embedded in Epon812 resin, followed by polymerization at 60 °C for 24 h. Ultra‐thin sections (70 nm) were made by a Reichert‐Jung ULTRACUT E type ultramicrotome and stained with lead citrate and uranyl acetate. Subsequently, the stained sections were examined using a transmission electron microscope (JEM1200, Japan).

### Immunofluorescence

SACC‐83 cells were seeded at a density of 2 × 10^4^ cells per well until a confluence of 70% was achieved. Then, cells were fixed with 4% paraformaldehyde at room temperature for 20 min, followed by permeabilization with 0.25% Triton X‐100 for 30 min. The cells were then blocked with 1% BSA at 37 °C for 30 min, followed by incubation with anti‐PRRX1 (1 : 250; Abcam) or anti‐Beclin‐1 (1 : 200; Proteintech) at 4 °C overnight. Subsequently, the cells were incubated with a FITC‐conjugated goat anti‐rabbit IgG (H + L) secondary antibody (1 : 100; Elabscience) for 1 h in the dark, followed by DAPI staining for 5 min. The cells were visualized using the Olympus FluoView™ confocal microscope.

### Gas chromatography mass spectrometry analysis (GC/MS)

Briefly, 10^6^ tumor cells were taken and resuspended in 0.2 mL PBS, and the ultrasonic probe was inserted into the PBS suspension for fragmentation. The cells were lysed in an ice bath for 1 min, and such procedure was repeated 4‐5 times. The cell homogenates were centrifuged at 10 800 × ***g*** for 15 min at 4 °C, and the supernatant was collected. Next, 1 mL 10% concentrated sulfuric acid‐methanol solution was added, and the mixture was methylated in a water bath at 70 °C for 1 h. After cooling, 1 mL n‐hexane was added to extract fatty acid methyl ester to the organic phase by oscillation, followed by the addition of 5 mL distilled water to wash sulfuric acid to the aqueous phase by oscillation, and it was allowed to stand for 2 h to absorb the upper organic phase. Subsequently, 0.4 µL of the methylated sample was taken for GC‐MS analysis. All analyses were performed in split mode (1 : 20) on an Agilent 7890a gas chromatograph connected to an Agilent 5975C Series MSD (Agilent Technologies, Santa Clara, CA, USA). The chromatographic columns were 30 m DB‐5 MS + DG capillary columns (5% phenyl, 95% dimethylpolysiloxane) with an internal diameter of 250 μm (Agilent Technologies) and a 25 m × 0.25 mm SLB‐IL82 column with a film thickness of 0.2 μm (Supelco). The injection volume was 1 μL. The MS source and MS quadrupole were maintained at 230 °C and 150 °C, respectively. The masses of the analytes were acquired in full‐scan mode with mass range of 30–650 m·z^−1^.

### FFA quantification

The contents of FFAs were determined using an FFA quantification colorimetric/fluorometric kit (BioVision, Waltham, MA, USA). The palmitic acid standard liquids in the kit were diluted to 0, 0.2, 0.4, 0.6, 0.8, or 1.0 nmol per well. Subsequently, 10 mg SACC tissue samples or 10^6^ SACC‐83 cells were homogenized with 200 µL chloroform‐Triton X‐100. The extracts were centrifuged and dried for 10 min. The dried lipids were dissolved in a 200 µL fatty acid assay buffer. The absorbance at a wavelength of 570 nm was determined for colorimetric assay, and Ex/Em = 535/590 nm was applied for fluorescence measurements in a microplate reader.

### Wound healing assay

SACC‐83 cells were seeded into six‐well plates at a density of 2.0 × 10^5^ cells per well, followed by serum‐free starvation for 1 day. A wound was created using a pipette tip, and the cells were cultured with the FBS‐free medium for 24 h. Wound closures were photographed, and the residual cell‐free region in wells was measured. The proportion of the cell‐free region was calculated and expressed as mean ± SD.

### Transwell invasion assay

The experiment was performed with 1.5 × 10^5^ cells and Matrigel‐coated membrane (24‐well insert, BD Biosciences; pore size, 8 μm). Migrated cells on the lower surface of the membrane were stained with crystal violet, and five randomly selected fields were counted.

### Statistical analysis

All statistical analyses were performed using the spss package (version 17.0). Student’s *t* test and analysis of variance (ANOVA) with Tukey’s post hoc tests were used for comparisons between two or multiple groups to determine significance, respectively. A value of *P* < 0.05 was considered statistically significant.

## Results

### PRRX1 expression is negatively associated with autophagy in SACC patients

**Fig. 1:**
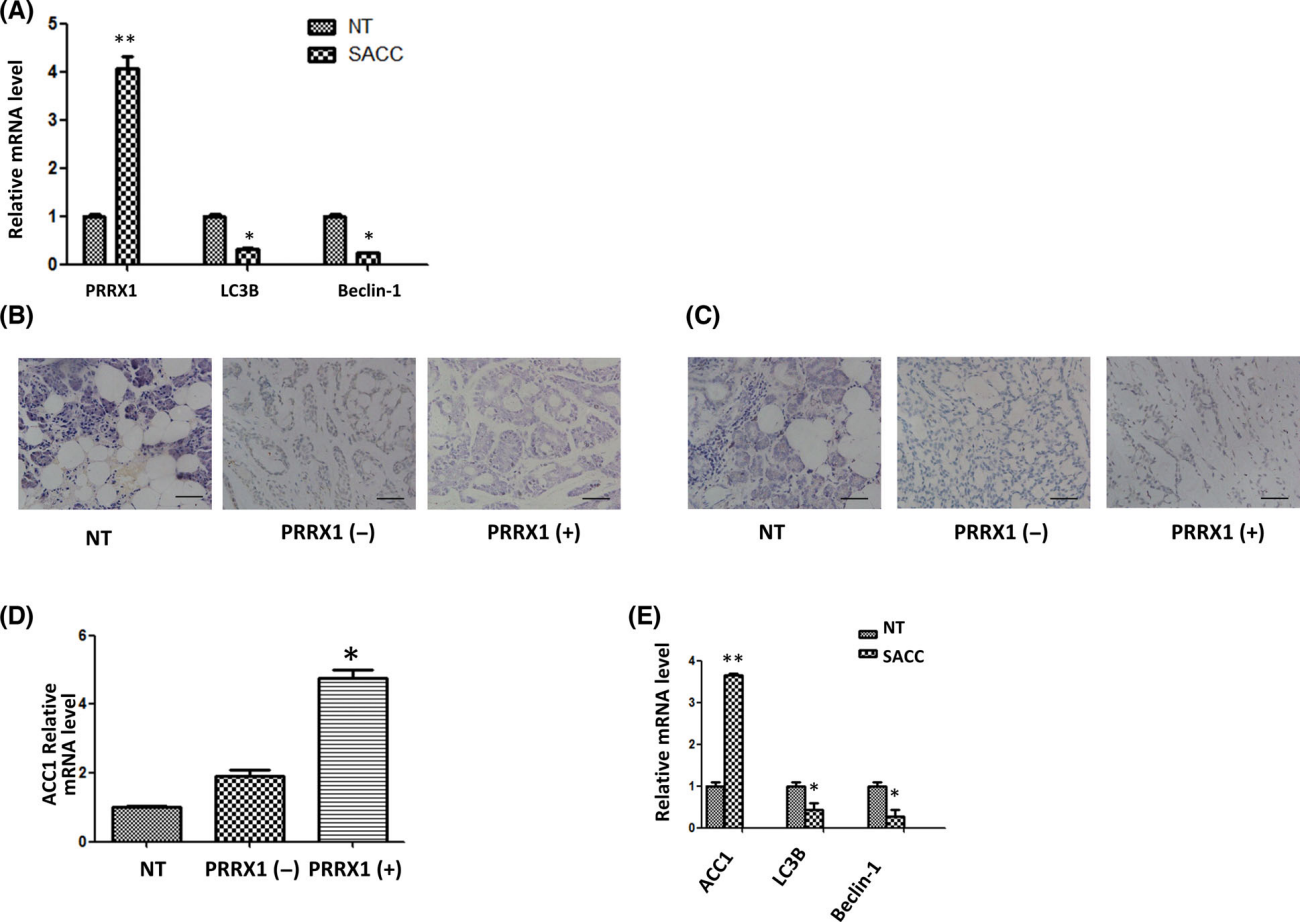
PRRX1 is significantly associated with autophagy in SACC patients. (A) RT‐qPCR analysis of the PRRX1 expression, and autophagic proteins LC3B and Beclin‐1 in SACC tissues. *P < 0.05, **P < 0.01 compared with normal tissues (NT). (B) Beclin‐1 and (C) ACC1 were detected in NT, PRRX1‐negative, and PRRX1‐positive SACC tissues by immunohistochemistry. Scale bar = 100 μm. (D) The expression of ACC1 in NT, PRRX1‐negative, and PRRX1‐positive SACC tissues by RT‐qPCR. *P < 0.05. (E) The relationship of ACC1 and LC3B/Beclin‐1. *P < 0.05, **P < 0.01 compared with NT. Error bars represent the mean ± SD of triplicate experiments (*n* = 3). Unpaired *t*‐test was performed to determine significance.

It is well known that autophagy has dual roles in cancer. However, its role in SACC remains largely unexplored. Our previous study has shown that the positive rate of PRRX1 in SACC tissues is remarkably greater compared with the normal salivary tissues (Fig. S2), and the poor prognosis in SACC patients is associated with the PRRX1 expression [21]. In the present study, the results of RT‐qPCR showed *PRRX1* was significantly enhanced, while *LC3B* and *BECLIN 1* were declined in SACC tissues compared with normal tissues (NT) of the periphery of benign salivary gland tumor (Fig. 1A). It showed the reversed interaction between PRRX1 and Beclin‐1 in SACC specimens by immunohistochemistry (Fig. 1B). Moreover, PRRX1 is known to induce the accumulation of fatty acid and insulin resistance in type 2 diabetes [20]. To identify its effect on the *de novo* synthesis of fatty acid in SACC, we found that the expression of ACC1 was positively correlated with PRRX1 in SACC specimens (Fig. 1C,D). To further identify the correlation between ACC1 and autophagy induced by PRRX1 in SACC, we conducted the correlation analyses of *ACC1* and *LC3B*/*BECLIN 1* in SACC specimens. There was a significant negative correlation between *ACC1* and *LC3B*/*BECLIN 1* expressions (Fig. 1E), suggesting that ACC1 could act to sequester autophagy in SACC tissues.

### PRRX1 suppresses autophagy, but fuels invasion and migration in SACC‐83 cells

**Fig. 2:**
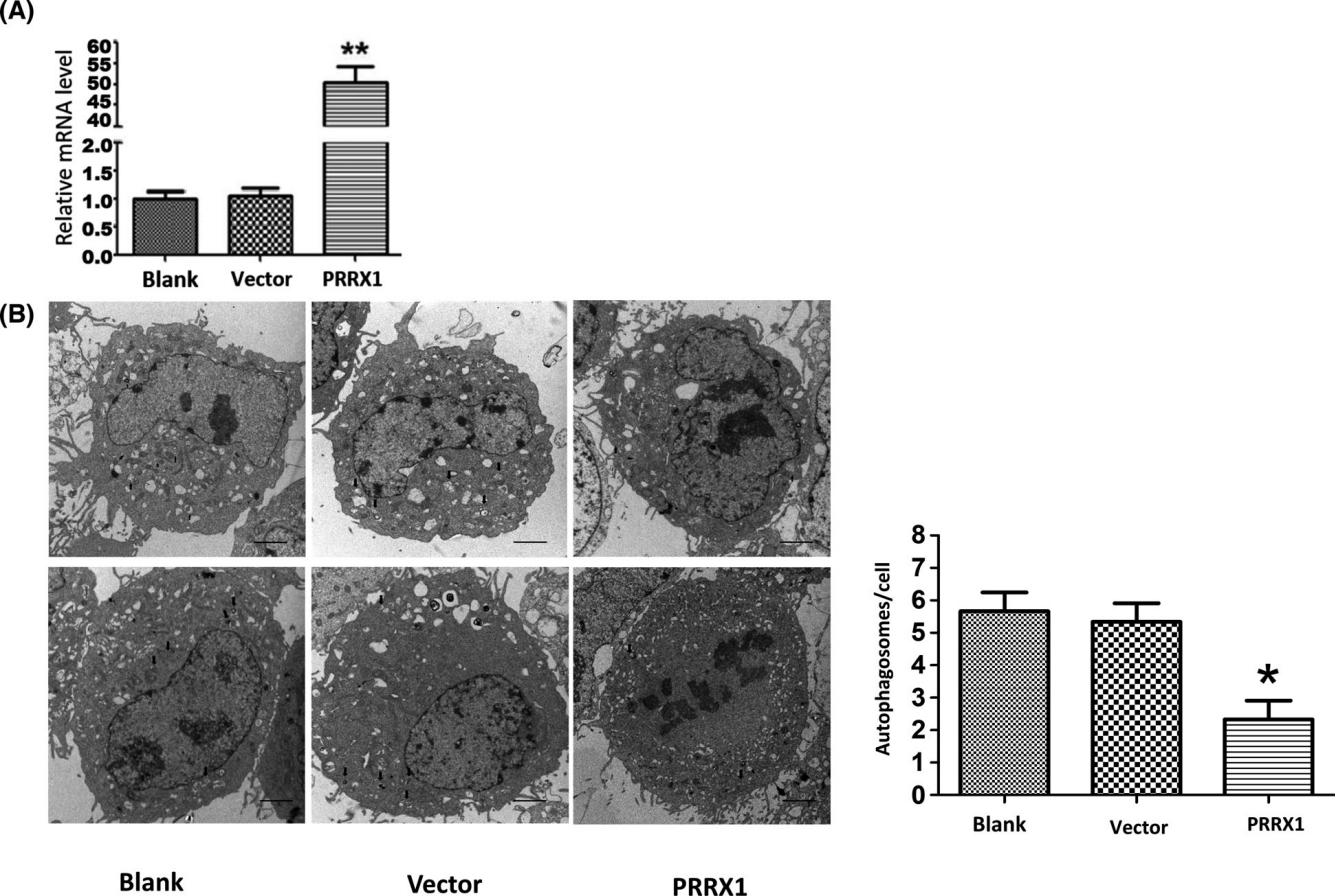
The number of autophagosomes is reduced after the lentiviral vector overexpressing PRRX1 is transfected into SACC‐83 cells. (A) PRRX1 was significantly upregulated after the lentiviral vector overexpressing PRRX1 was transfected into SACC‐83 cells. *P < 0.05, **P < 0.01. (B). The number of autophagosomes was decreased in the PRRX1 overexpression group. Black arrows represent autophagosomes. Scale bar = 2 μm. Error bars represent the mean ± SD of triplicate experiments (*n* = 3). One‐way ANOVA statistical analysis was performed to determine significance.

**Fig. 3:**
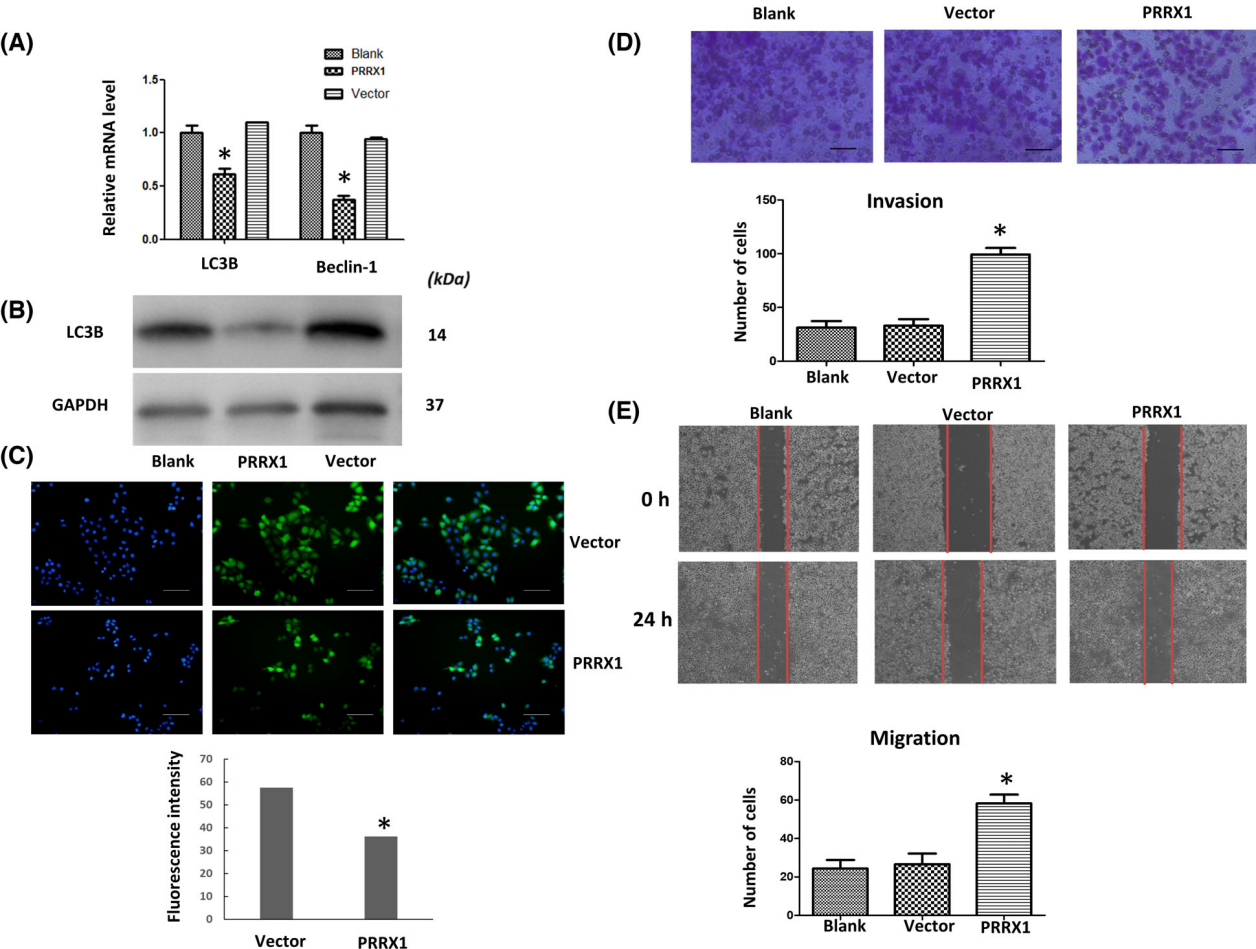
PRRX1 inhibits autophagy‐associated proteins but facilitates invasion and migration in SACC‐83 cells. LC3 and Beclin‐1 were significantly suppressed by (A) RT‐qPCR and (B) western blotting analysis. *P < 0.05. (C) Beclin‐1 was downregulated in the PRRX1 overexpression group by immunofluorescence. Scale bar = 100 μm. (D) invasion and (E) migration were enhanced. Scale bar = 100 μm. Error bars represent the mean ± SD of triplicate experiments (*n* = 3). One‐way ANOVA statistical analysis was performed to determine significance.

To determine the impacts of PRRX1 on autophagy, *PRRX1* overexpression was achieved in SACC‐83 cells, which was confirmed by RT‐qPCR (Fig. 2A). Next, TEM showed that the number of autophagosomes was reduced in SACC‐83 cells overexpressing PRRX1 (Fig. 2B). Moreover, the expressions of LC3B and Beclin‐1 were significantly decreased in the PRRX1‐overexpressing group (Fig. 3A–C. Fig. 3D–3E indicates that the invasion and migration capabilities of SACC‐83 cells overexpressing PRRX1 were increased.

### PRRX1 activating ACC1‐induced FFA reprogramming triggers autophagy in SACC‐83 cells

**Fig. 4:**
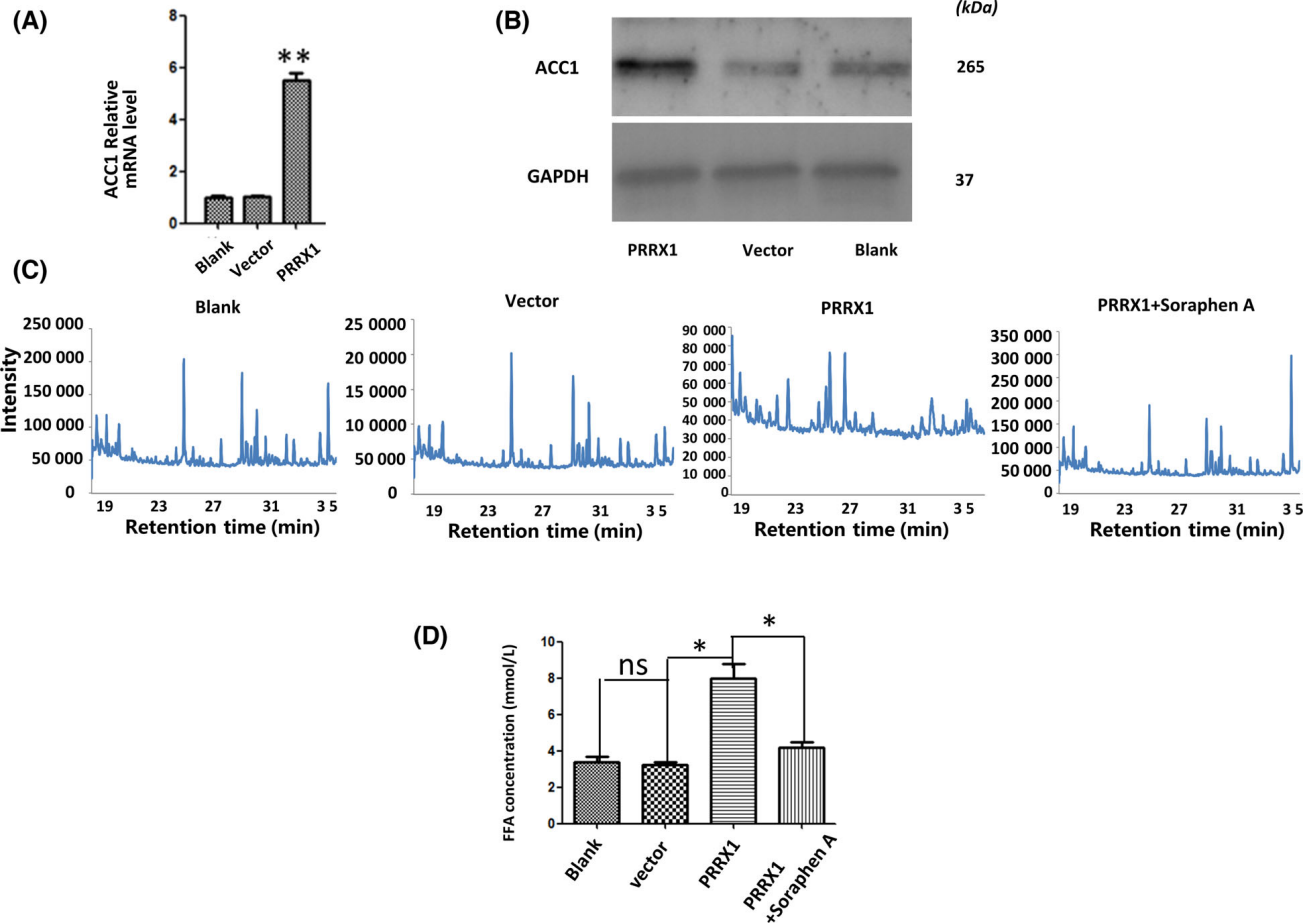
ACC1 stimulates FFA reprogramming in response to autophagy in PRRX1‐overexpressing cells. ACC1 was significantly elevated by (A) RT‐qPCR and (B) western blotting analysis. **P < 0.01. (C) The gas chromatography mass spectrometry chromatograms of FFAs in PRRX1‐overexpressing SACC‐83 cells after Soraphen A treatment. (D) The FFA level in PRRX1‐overexpressing SACC‐83 cells after Soraphen A treatment by FFA quantification colorimetric/fluorometric kit. *P < 0.05. Error bars represent the mean ± SD of triplicate experiments (*n* = 3). One‐way ANOVA statistical analysis was performed to determine significance.

**Fig. 5:**
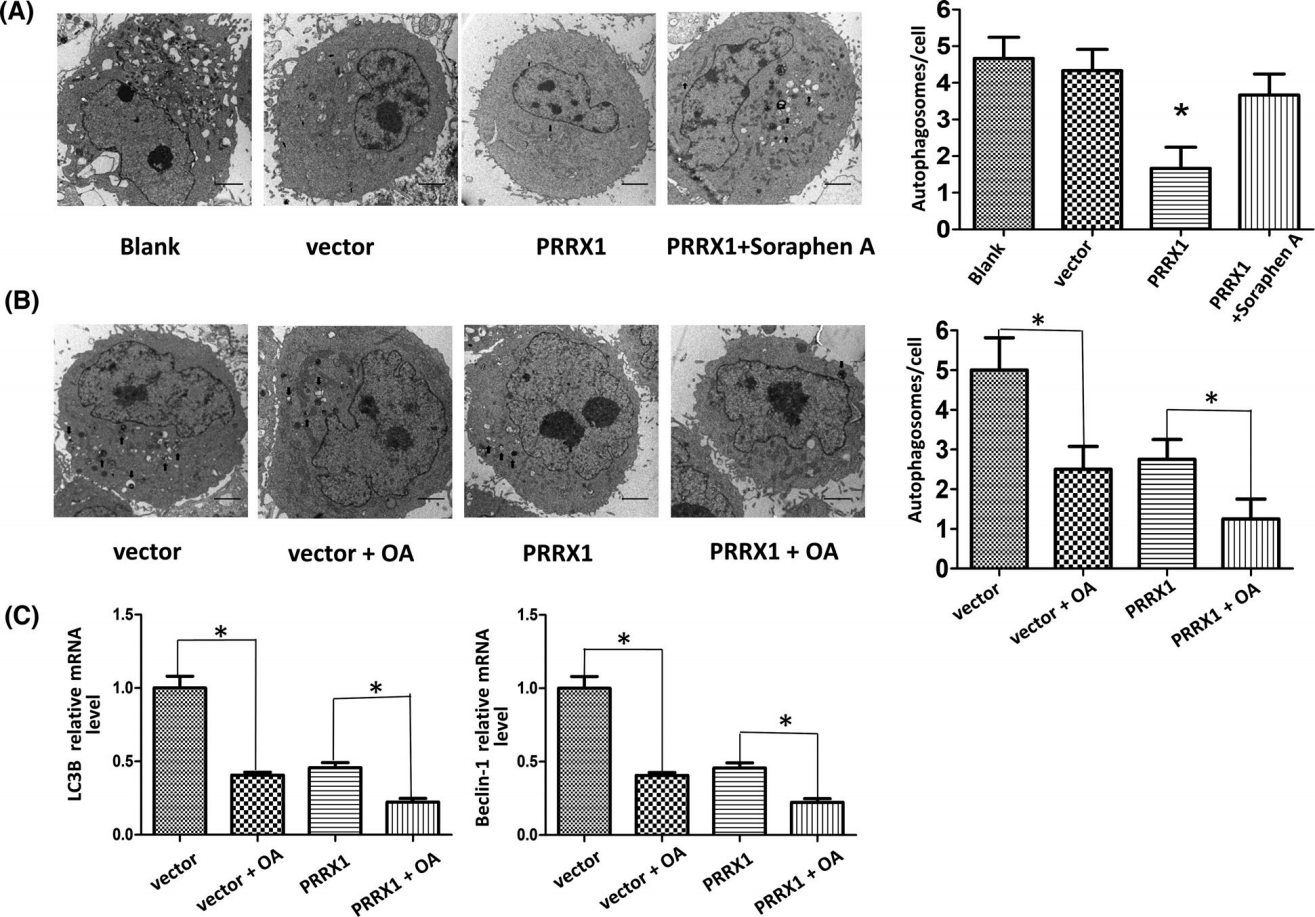
(A) The number of autophagosomes was increased in PRRX1‐overexpressing SACC‐83 cells after Soraphen A treatment. Black arrows represent autophagosomes. *P < 0.05. Scale bar = 2 μm. (B) The number of autophagosomes was decreased in the vector and PRRX1 overexpression group treated with OA in SACC‐83 cells. Black arrows represent autophagosomes. *P < 0.05. Scale bar = 2 μm. (C) OA significantly suppressed autophagy‐associated proteins LC3B and Beclin‐1 by RT‐qPCR in the vector and PRRX1 overexpression group. *P < 0.05. Error bars represent the mean ± SD of triplicate experiments (*n* = 3). One‐way ANOVA statistical analysis was performed to determine significance.

Previous investigations have indicated that the level of autophagy highly depends on ACC1 in yeast [29]. Additionally, as a key enzyme of the *de novo* fatty acid synthesis, ACC1 has regulatory effects on fatty acid metabolism in prostate cancer, hepatocellular carcinoma, etc. Therefore, we examined whether there was an association between the PRRX1 and ACC1 expressions. We found that the expression of ACC1 was dramatically enhanced in PRRX1‐overexpressing SACC‐83 cells (Fig. 4A,B). Moreover, we further determined whether ACC1 affected fatty acid programming and autophagy in PRRX1‐overexpressing SACC‐83 cells utilizing the ACC1 inhibitor, Soraphen A. We found that the constituent ratio of FFA was decreased and the proportion of long‐chain FFAs more than 16C was reduced after Soraphen A treatment compared with the PRRX1‐positive group (Fig. 4C, Table 1). The quantitative analysis of FFAs revealed that the higher FFA levels in PRRX1‐overexpressing SACC‐83 cells were remarkably reversed (Fig. 4D). Meanwhile, the number of autophagosomes was rescued by Soraphen A treatment in the PRRX1‐overexpressing group (Fig. 5A). Subsequently, to determine whether FFA could compromise autophagy, SACC‐83 cells were treated with Oleic acid (OA, a kind of long‐chain fatty acid) coupled to BSA, which was processed as OA solution as described previously [30, 31]. The results showed that the number of autophagosomes was reduced and the expressions of *LC3B* and *Beclin‐1* were significantly inhibited when SACC‐83 cells were stimulated with OA in vector and PRRX1‐overexpressing groups (Fig. 5B,C).

**Table 1:** FFA composition of the PRRX1‐overexpressed SACC‐83 cells after Soraphen A treatment (%, *n* = 3).

FFA composition	Blank	Vector	PRRX1	PRRX1+Soraphen A
C14:0	81.85 ± 5.46	70.63 ± 4.12	1.17 ± 0.11	67.55 ± 4.42
C16:0	18.15 ± 1.06	29.37 ± 1.34	39.67 ± 2.82	21.86 ± 1.85
C16:1	0		8.36 ± 0.60	0
C17:0	0		11.80 ± 0.94	0
C18:0	0	0	0	10.59 ± 1.01
C18:1	0		26.35 ± 1.43	0
C18:2	0		12.65 ± 1.27	0

## Discussion

To fulfill the rapid cell proliferation and metastasis, lipid, such as fatty acid, plays a critical role in tumor cells [32]. Malonyl‐CoA synthesized from acetyl‐CoA, helped along by the catalytic action of ACC, is the rate‐limiting step in the synthesis of FFA. Furthermore, malonyl‐CoA, serving as a substrate, can aid for acyl chain elongation in process of FFA synthase and also regulate fatty acid β‐oxidation via suppressing carnitine palmitoyltransferase I [33]. Accumulating evidence has indicated that ACC overexpressed in human cancers, such as prostate carcinoma, liver carcinoma, and ductal carcinoma and lobular carcinoma in situ of breast, can fuel growth and development of cancer cells [34, 35, 36, 37, 38, 39]. ACC has an unusual expression in SACC cells [40]. ACC1 inhibitors impair tumor metastasis and invasion by reducing the formation of invadopodia and altering the FFA synthesis pathway [41, 42]. Moreover, inactivated ACC and stimulated autophagy can impede lipogenesis and lipid accumulation [43]. Besides, autophagy can be blockaded via modulation of AMPK/ACC signaling pathway, while phosphorylation of AMPK/ACC can suppress lipogenesis in hepatocellular carcinoma [44, 45].

As one member of the homeobox protein family, PRRX1 plays a role in transcriptional activation and induction of downstream genes through the transcriptional activation of growth factors or other effect factors [46]. Our previous results have demonstrated that PRRX1 induces EMT and fuels the reprogramming of FFA metabolism. However, the exact underlying mechanism remains largely unexplored. As a rate‐limiting enzyme in *de novo* synthesis of fatty acid, ACC1 induces the reprogramming of fatty acid metabolism [47]. Our study proved that the expression of ACC1 was remarkably upregulated in the PRRX1‐overexpressing group. Meanwhile, the levels and constituent ratio of FFAs were increased, and the proportion of long‐chain FFAs more than 16C was increased. However, when we added ACC1 inhibitor (Soraphen A) to the SACC cells overexpressing PRRX1, the FFA levels and the constituent ratio of FFAs were reduced, and the proportion of long‐chain FFAs more than 16C was decreased compared with the PRRX1‐positive group. Therefore, PRRX1 overexpression could upregulate fatty acid metabolism via ACC1.

Previous studies have found that autophagy may suppress metastasis in carcinomas [48, 49]. Chenxi Li has found that the expressions of markers for autophagy (*LC3* and Beclin‐1) are downregulated in highly metastatic SACC or late TNM stages [23]. According to our study, there was a negative correlation between *ACC1* and *LC3B*/*BECLIN 1* expression in SACC specimens. We found that LC3B/Beclin‐1 expression was reduced with the significant upregulation of ACC1 and FFA in PRRX1‐overexpressing SACC cells. Meanwhile, the number of autophagosomes was also decreased, which was rescued by ACC1 inhibitor treatment. Moreover, the metastasis and invasion capabilities of SACC cells were also enhanced with the overexpression of PRRX1. Collectively, PRRX1 activated the ACC1‐induced metabolic reprogramming of FFA to facilitate the invasion and metastasis by reducing autophagy in SACC. Furthermore, PRRX1 might be used as a potential marker for invasion and metastasis of SACC and the target for clinical therapy.

## Conflict of interest

The authors declare no conflict of interest.

## Author contributions

YPJ contributed to conceive and design the study; JG, KJM, and LZ performed the experiments and collected the data; JG and KJM wrote the manuscript; TL performed statistical analyses and polished the language; and BDZ and YPJ provided technical and economical support to the project. All authors have read and approved the manuscript.

## Supporting information


**Fig S1**. The cell STR reports of SACC‐83.

**Fig S2**. (A) The low expression of PRRX1 in normal salivary gland tissue. (B) The low expression of PRRX1 in SACC sample. (C)The high expression of PRRX1 in SACC sample. Scale bar = 100 μm.

**Table S1**. STR profiles of SACC‐83.

## Data Availability

The data that support the findings of this study are available from the corresponding author [jiangyaping@qdu.edu.cn] upon reasonable request.
