# Supplementary figures and images for: Paired related homeobox 1 attenuates autophagy via acetyl‐CoA carboxylase 1‐regulated fatty acid metabolism in salivary adenoid cystic carcinoma
Source: FEBS Open Bio. 2022 Mar 29;12(5):1006–16. doi: 10.1002/2211-5463.13367 (PMC9063443; doi:10.1002/2211-5463.13367)

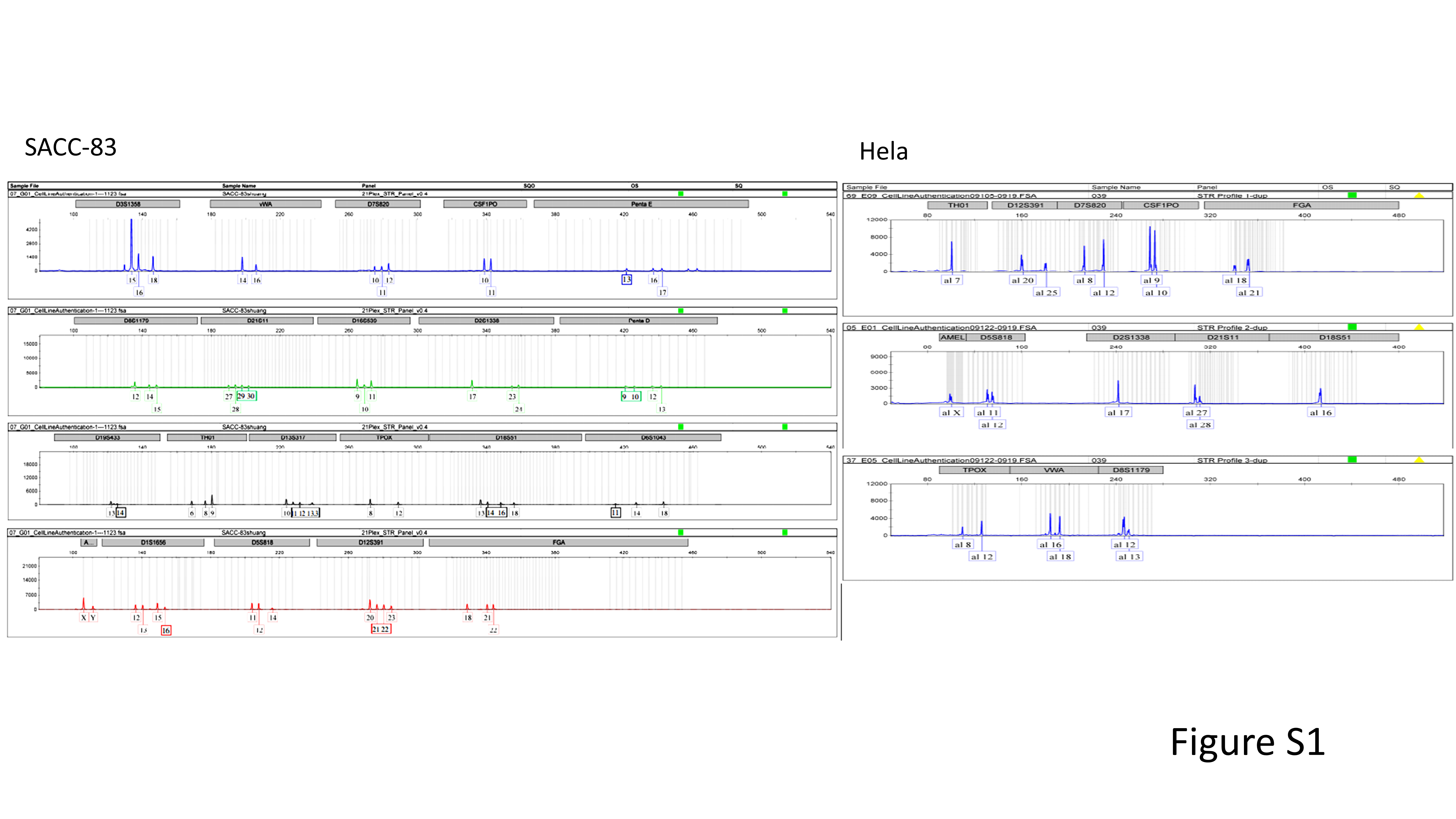

Supplement: Supplementary file 1 — Fig S1. The cell STR reports of SACC‐83. [file FEB4-12-1006-s003.tif]

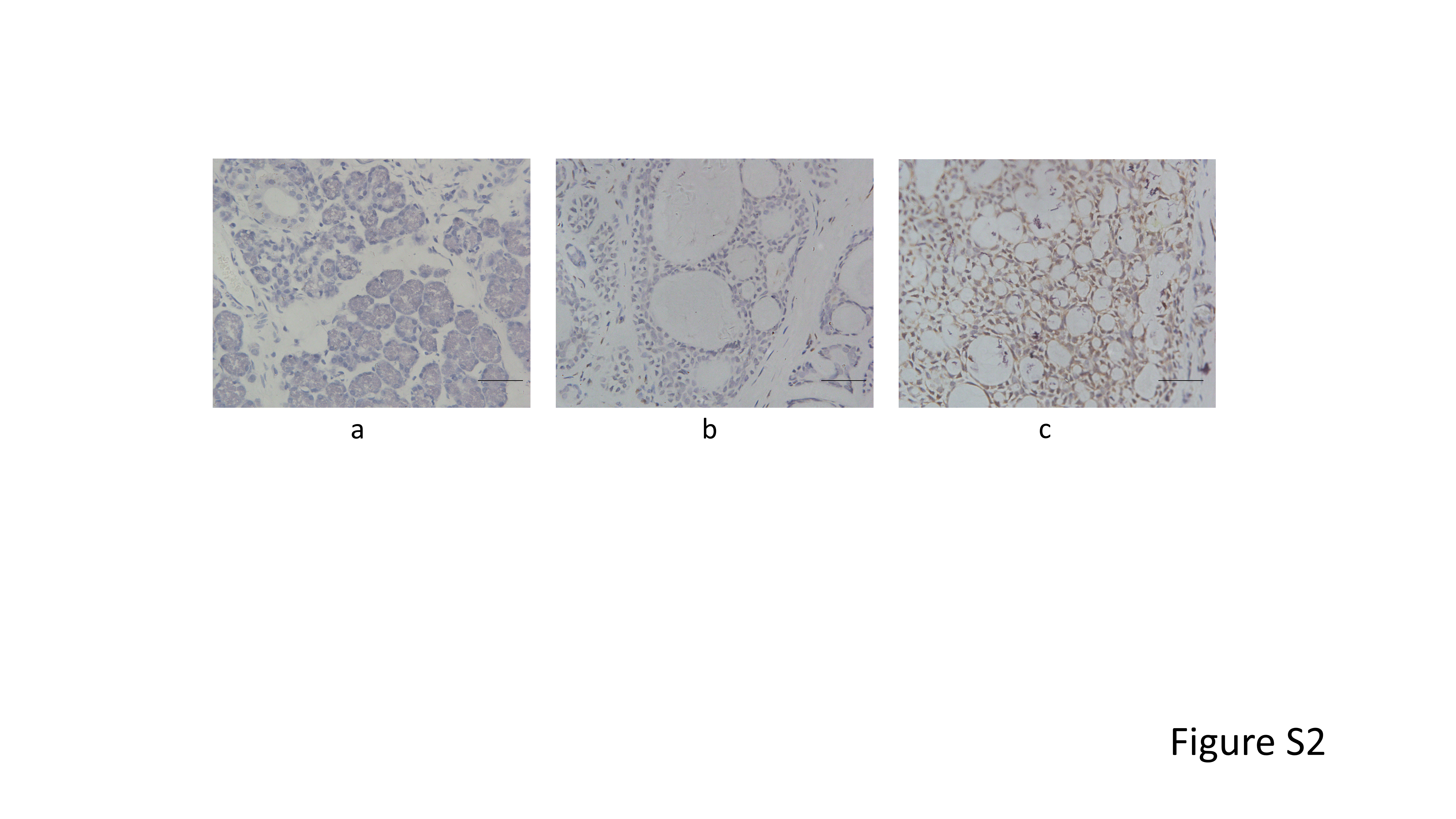

Supplement: Supplementary file 2 — Fig S2. (A) The low expression of PRRX1 in normal salivary gland tissue. (B) The low expression of PRRX1 in SACC sample. (C)The high expression of PRRX1 in SACC sample. Scale bar = 100 μm. [file FEB4-12-1006-s002.tif]
