# Supplementary material for: Paired related homeobox 1 attenuates autophagy via acetyl‐CoA carboxylase 1‐regulated fatty acid metabolism in salivary adenoid cystic carcinoma
Source: FEBS Open Bio. 2022 Mar 29;12(5):1006–16. doi: 10.1002/2211-5463.13367 (PMC9063443; doi:10.1002/2211-5463.13367)
Supplement: Supplementary file 3 — Table S1. STR profiles of SACC‐83. [file FEB4-12-1006-s001.docx]

| Markers | SACC-83 | HeLa |
| --- | --- | --- |
| D19S433 | 13, 14 | 13, 14 |
| D5S818 | 11,12,14 | 11, 12 |
| D21S11 | 27,28,29 | 27, 28 |
| D18S51 | 13,14,16,18 | 16, 16 |
| PentaD | 9, 10,12,13 | 8, 15 |
| D3S1358 | 15, 16, 18 | 15, 18 |
| D13S317 | 10,11,12,13.3 | 12, 14 |
| D7S820 | 10, 11, 12 | 8, 12 |
| D16S539 | 9,10,11 | 9, 10 |
| CSF1PO | 10, 11 | 9, 10 |
| D2S1338 | 17,23,24 | 17, 17 |
| Amelo genin | X, Y | X, X |
| vWA | 14, 16 | 16, 18 |
| D8S1179 | 12,14,15 | 12, 13 |
| D12S391 | 20,21,22,23 | 20, 25 |
| FGA | 18,21,22 | 18, 21 |
| TH01 | 6,8,9 | 7, 7 |

**Table S1 STR profiles of SACC-83**
